# A234 ELEVATED DIRECT BILIRUBIN DOES NOT PREDICT ELEVATED CONJUGATED BILIRUBIN IN NEONATES

**DOI:** 10.1093/jcag/gwae059.234

**Published:** 2025-02-10

**Authors:** P Samaraweera, H Brill

**Affiliations:** William Osler Health System, Brampton, ON, Canada; William Osler Health System, Brampton, ON, Canada

## Abstract

**Background:**

Neonatal cholestasis guidelines consider direct and conjugated bilirubin to be equivalent because the delta bilirubin component of direct bilirubin is assumed to be negligible. Since 2015 William Osler Health System automatically measures conjugated bilirubin if the direct bilirubin is 20 µmol/L or greater in patients under 3 months old. This afforded us the opportunity to assess if direct bilirubin accurately reflects conjugated bilirubin.

**Aims:**

To determine if direct bilirubin reliably reflects conjugated bilirubin levels in neonates and confirms the appropriateness of further investigation of neonatal cholestasis

**Methods:**

Patient data from 2015 to 2024 was retrospectively collected and analyzed, focusing on neonates three months or younger with direct bilirubin levels of 20 µmol/L or higher. Both Direct and Conjugated bilirubin were measured using different assays. Main outcome measure was the difference between direct and conjugated bilirubin measurements.

**Results:**

Results: 121 subjects were identified. Average direct bilirubin (32.0µmol/L) and average conjugated bilirubin (8.9µmol/L) are significantly different (p=0.008). A direct bilirubin below 22 µmol/L almost never results in a conjugated bilirubin above 2 µmol/L. Moreover, for a conjugated bilirubin of 17 or higher (the guideline trigger for neonatal cholestasis investigations), the lowest direct bilirubin measured was 34.

**Conclusions:**

Direct Bilirubin is not an analogous proxy for conjugated bilirubin. Future guidelines should be modified to require confirming cholestasis with a conjugated bilirubin if direct bilirubin is the default tes

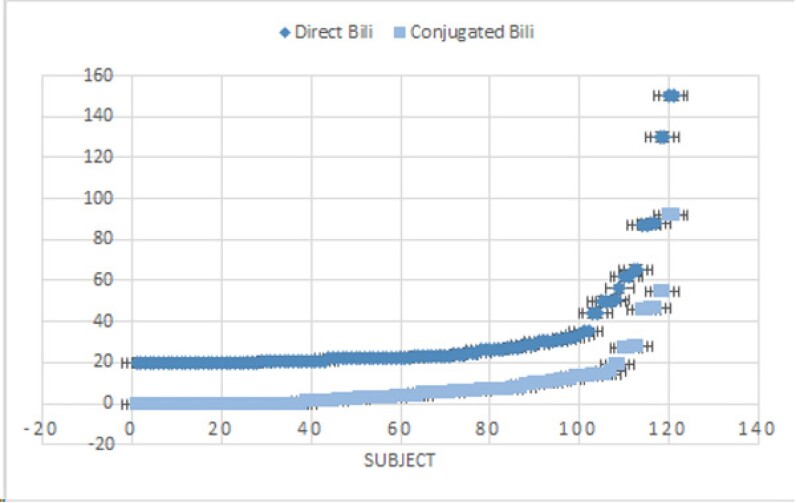

**Funding Agencies:**

None

